# Treatment strategies and treatment-related adverse events in MG according to the age of onset

**DOI:** 10.3389/fneur.2024.1277420

**Published:** 2024-03-11

**Authors:** João Moura, Joana Fernandes, Maria João Lima, Ana Paula Sousa, Raquel Samões, Ana Martins Silva, Ernestina Santos

**Affiliations:** ^1^Department of Neurology, Centro Hospitalar Universitário de Santo António, Porto, Portugal; ^2^Unit of Multidisciplinary Research in Biomedicine (UMIB), Instituto de Ciências Biomédicas Abel Salazar (ICBAS), University of Porto, Porto, Portugal; ^3^Department of Neurology, Unidade Local de Saúde de Matosinhos, Porto, Portugal; ^4^Department of Neurophysiology, Hospital de Santo António, Centro Hospitalar Universitário Do Porto, Porto, Portugal; ^5^Laboratory for Integrative and Translational Research in Population Health (ITR), Porto, Portugal

**Keywords:** myasthenia gravis, late-onset, comorbidities, steroid-sparing, immunosuppression

## Abstract

**Introduction:**

Early-onset (EOMG) and late-onset (LOMG) are distinct groups of MG patients. It is unclear if treatment strategies and treatment-related adverse events may differ according to the age of MG onset.

**Methods:**

This single-center retrospective study includes all MG patients followed at a tertiary center since 2007. We reviewed the electronic clinical records.

**Results:**

In total, 212 patients were identified, 142 (67.0%) females, with a median disease duration of 10 years. The median age of symptom onset was 42.0 (26.0–64.5) years, with 130 (61.3%) EOMG cases and 82 (38.7%) LOMG. EOMG were more frequently female, had longer disease duration and often more generalized MG (*p* < 0.001). Comorbidities were significantly more frequent in LOMG (67.1%) compared to EOMG (53.1%) (*p* = 0.002). Steroid-related adverse effects motivating the switch to steroid-sparing agents (82.0%) were different between groups, with hypertension, hypercholesterolemia, diabetes mellitus and malignancies being more common in LOMG. At the same time, osteoporosis and dyspepsia were more frequent in EOMG (*p* < 0.001). The most common first-line choice was azathioprine (45.8%), and rituximab was used in 4 patients (1.9%).

**Conclusion:**

Our study shows that treatment modalities are similar between EOMG and LOMG, while steroid-related adverse events appear to be distinct.

## Introduction

Myasthenia gravis (MG) is a chronic autoimmune neuromuscular disease characterized by ocular and generalized muscle weakness. This disorder has a heterogeneous pathogenesis and variable phenotype associated with distinct disease subtypes (1, 2). When considering the age of onset, patients <50 years are considered early-onset MG (EOMG), while patients with disease onset above 50 years belong to the late-onset MG group (LOMG).

Classically, treatment response is considered satisfactory in MG. The approval of novel immunosuppressive drugs to treat MG cases offers the opportunity to tailor the treatment to each patient. Baseline comorbidities and treatment-related side effects are essential aspects to consider in this process (3). Some studies suggest that EOMG and LOMG may differ in these characteristics (4, 5). However, it is still unclear if treatment-related adverse events are associated with the age of MG onset.

This study aims to describe a MG cohort and evaluate comorbidities, treatment strategies and treatment-related adverse events between EOMG and LOMG.

## Methods

We retrospectively analyzed the medical records of MG patients from an institutional database that contains all MG cases followed in the Centro Hospitalar Universitario de Santo António Neuroimmunology Outpatient Clinic. This database is updated annually since 2007. MG was diagnosed by practicing neurologists specialized in neuroimmunology (ES, AMS, APS, and RS) based on a combination of clinical features, neurophysiological studies, antibody testing and response to pyridostigmine (2). Patients aged <50 years were classified as EOMG, while patients ≥50 years were considered LOMG (1).

We collected information concerning sex, age of onset and age at diagnosis. We extracted data on the prevalence of different comorbidities using what was defined in previous studies. Comorbidities were classified as treatment-related if they appeared after the initiation of a specific treatment and if the neurologist considered the comorbidity an effect of treatment, according to the medical records. The baseline Myasthenia Gravis Foundation of America (MGFA) score and the occurrence of myasthenic crisis or hospital admission due to MG during the disease course were retrospectively collected. Data on the use of anticholinesterases (and maximum dose), steroids (and maximum dose), and other immunosuppressors used during the disease course was additionally detailed.

Qualitative variables were studied using absolute and relative frequencies. The median and interquartile range (p25–p75) (IQR) were calculated for quantitative variables. An *X*^2^ was used to compare categorical variables, while a Mann–Whitney U test was used for continuous variables. Statistical analysis was performed in SPSS Statistics version 29. A *p*-value < 0.05 was considered statistically significant.

This study was approved by Centro Hospitalar Universitário de Santo António Ethical Committee. Informed consent was waived due to the retrospective nature of the study.

## Results

**Figure 1:**
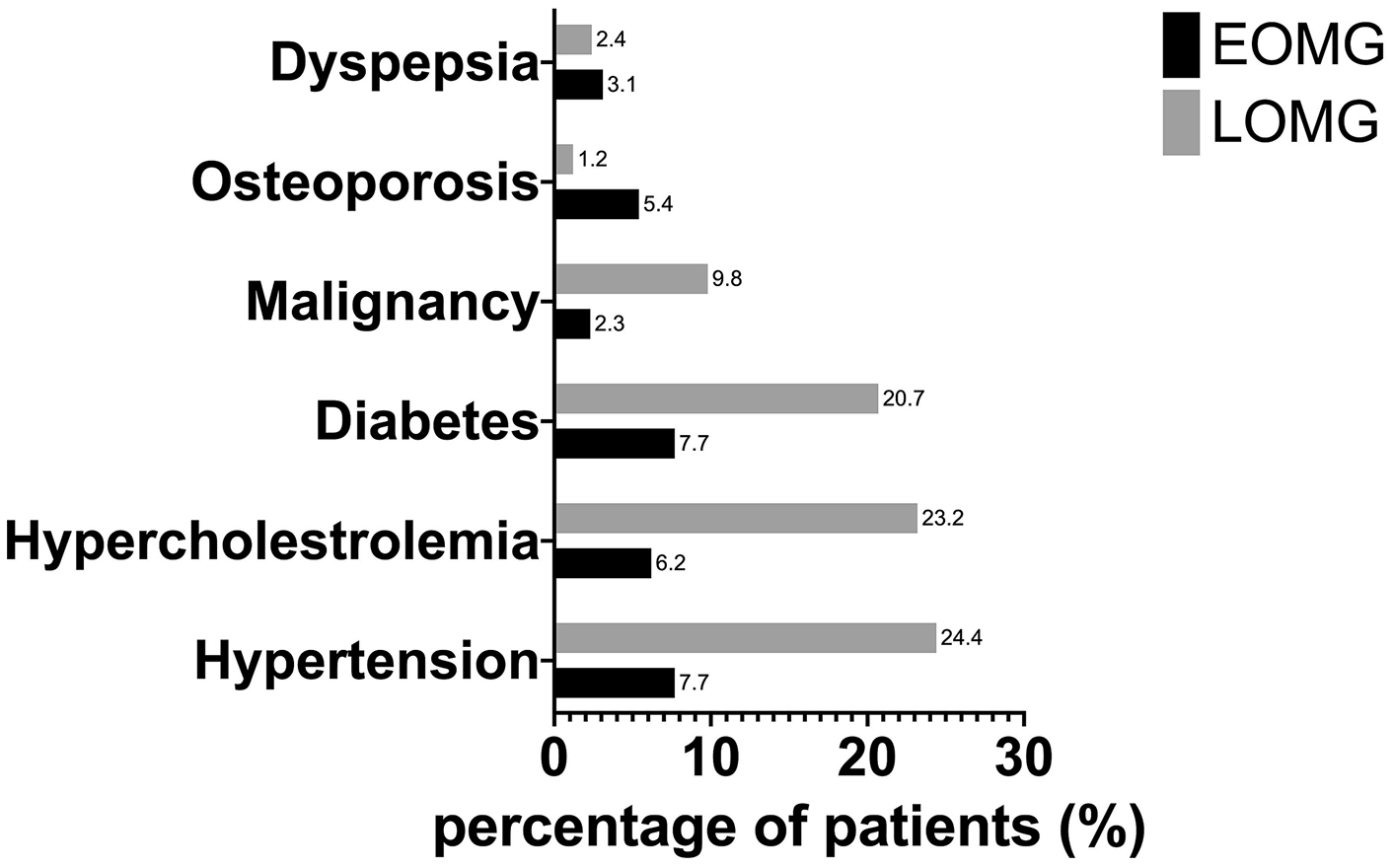
Distribution of MGFA scores according to the age of onset.

In total, 212 patients were identified, 103 (79.2%) female. The median disease duration was ten years, and the median follow-up time was 8 years. The median age of symptom onset was 42.0 (26.0–64.5) years, with 130 (61.3%) EOMG cases and 82 (38.7%) LOMG. Table 1 summarizes the characteristics of the cohort. Overall, EOMG were more frequently female and had longer disease duration. MG phenotypes were significantly different between groups, with EOMG patients having significantly more generalized MG (80.0% vs. 56.1%) and LOMG patients having more ocular MG (43.9% vs. 20.0%) (*p* < 0.001). Figure 1 shows the distribution of baseline MGFA scores concerning the age of onset.

**Table 1:** Characterization of the MG cohort according to age of onset.

Variable	EOMG 130 (61.3%)	LOMG 82 (38.7%)	*p*
Female, *n* (%)	103 (79.2)	39 (47.6)	<0.001
Disease duration, median (IQR)	28 (15.0–39.0)	7.0 (4.0–13.0)	<0.001
**Disease type, *n* (%)**			
Ocular	26 (20.0)	36 (43.9)	<0.001
Generalized	104 (80.0)	46 (56.1)	
**Antibodies, *n* (%)**			
Anti-AchR	84 (64.6)	51 (62.2)	0.878
Anti-MuSK	9 (6.9)	2 (2.4)	0.497
Anti-titin	7 (5.4)	19 (23.2)	<0.001
Thymoma, *n* (%)	17 (13.1)	13 (15.9)	<0.001
Deceased	2 (1.5)	16 (19.5)	0.002
Number of comorbidities, median, IQR	1 (0.0–1.0)	1.0 (0.0–3.0)	0.002
**Comorbidities, *n* (%)**			
HTA	10 (7.7)	25 (30.5)	<0.001
Hypercholestrolemia	9 (6.9)	22 (26.8)	<0.001
Diabetes	14 (10.8)	20 (24.4)	0.012
Other cancers	12 (9.2)	12 (14.6)	0.268
Cataracts	8 (6.2)	4 (4.9)	0.770
Osteoporosis	7 (5.4)	2 (2.4)	0.487
Oportunistic infections	5 (3.9)	6 (7.3)	0.343
Dyspepsia	4 (3.1)	2 (2.4)	1.000
PVT/PE	0	4 (4.9)	0.021
Thyroid disease	7 (5.4)	6 (7.3)	0.570
Other autoimmune disease	26 (20.0)	6 (7.3)	0.031
Ashtma	5 (3.9)	4 (4.9)	0.737
Psoryasis	3 (2.3)	0	0.285
Neuromyotonia	4 (3.1)	0	0.160
Coronary disease	8 (6.2)	5 (6.1)	1.000
Atrial Fib	3 (2.3)	4 (4.9)	0.434
Prostate hyperplasia	0	2 (2.4)	0.148
Depression	4 (3.1)	3 (3.7)	1.000
Sleep apnea	5 (3.8)	1 (1.2)	0.409
Stroke/TIA	3 (2.3)	2 (2.4)	1.000
CKD	4 (3.1)	5 (6.1)	0.313
PVD	1 (0.8)	1 (1.2)	1.000
COPD	0	1 (1.2)	0.387
HF	2 (1.5)	2 (2.4)	0.641
Hypoacusis	0	2 (2.4)	0.148
Chronic liver disease	1 (0.8)	1 (1.2)	1.000
Headache	1 (0.8)	0	1.000
Bronchiectasis	1 (0.8)	0	1.000
Dementia	1 (0.8)	3 (3.7)	0.301
Gout	0	2 (2.4)	0.148
Epilepsy	0	1 (1.2)	0.387
PD	1 (0.8)	3 (3.7)	0.301
Alcoholism	1 (0.8)	1 (1.2)	1.000
Psychosis	0	1 (1.2)	0.387
**Treatment**			
Piridostigmine ever, *n* (%)	127 (97.7)	72 (87.8)	0.024
Piridostigmine dose, median (IQR)	300.0 (240.0–360.0)	240.0 (180.0–300.0)	<0.001
Steroids ever, *n* (%)	103 (79.2)	55 (67.1)	0.219
Steroids dose, median (IQR)	40.0 (20.0–60.0)	30.0 (20.0–52.5)	0.118
Immunoglobulin during acute phase, *n* (%)	43 (33.1)	22 (26.9)	0.328
Plasmapheresis during acute phase, *n* (%)	8 (6.2)	5 (6.1)	0.599
Thymectomy, *n* (%)	66 (50.8)	15 (18.3)	<0.001
Steroid sparing, first line, *n* (%)			0.074
AZA	69 (53.1)	28 (34.1)	
MMF	5 (3.8)	3 (3.7)	
RTX	3 (2.3)	1 (1.2)	
MTX	4 (3.1)	4 (4.9)	
CYC	0	1 (1.2)	
Steroid sparing, second line, *n* (%)			0.068
MTX	18 (13.8)	8 (9.8)	
MMF	16 (12.3)	5 (6.1)	
RTX	5 (3.8)	0	
AZA	3 (2.3)	1 (1.2)	
CYC	1 (0.8)	0	
Steroid sparing, third line, *n* (%)			0.042
RTX	9 (6.9)	4 (4.9)	
MMF	6 (4.6)	0	
MTX	3 (2.3)	0	
CYC	1 (0.8)	0	
Efgartigimod	1 (0.8)	0	
Tacrolimus	1 (0.8)	0	

AZA, azathioprine; MMF, mycophenolate mofetil; MTX, methotrexate; RTX, rituximab; CYC, cyclophosphamide; IQR, Interquartile range; PVT, Peripheral venous thrombosis; PE, pulmonary embolism; CKD, chronic kidney disease; COPD, chronic obstructive pulmonary disease; HF, heart failure; PD, Parkinson’s Disease.

At least one comorbidity was present in 124 patients (58.5%), with a median of 1.0 (0.0–2.0) comorbidity per patient. Comorbidities were significantly more frequent in LOMG (67.1%) compared to EOMG (53.1%) (*p* = 0.002). In particular, arterial hypertension, dyslipidemia, diabetes mellitus and venous thromboembolism were significantly more associated with LOMG, as shown in Table 1. Patients with and without thymomas had a similar number of comorbidities (*p* = 0.582).

**Figure 2:**
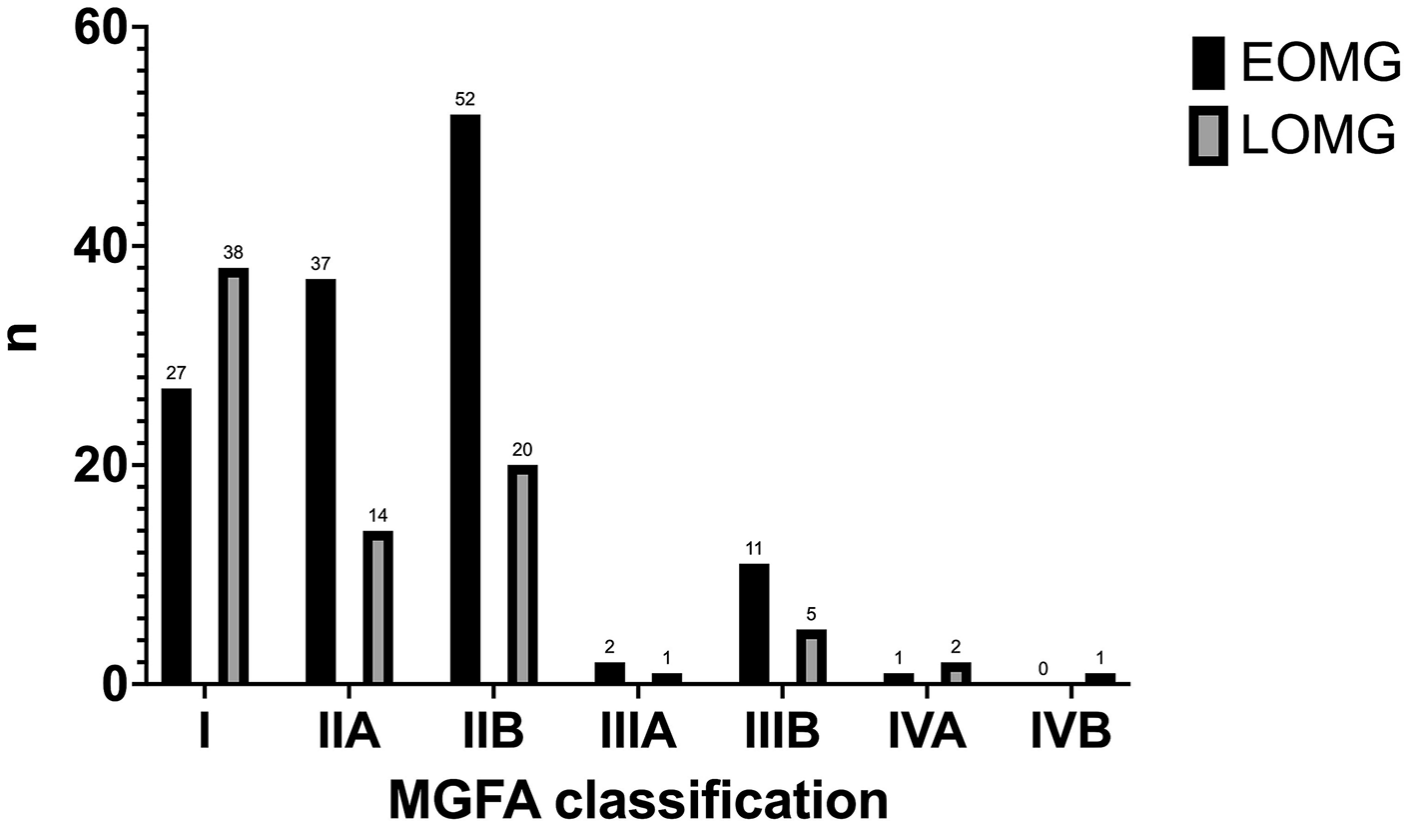
Adverse effects associated with steroids in EOMG and LOMG.

Fifty patients (23.6%) had at least one myasthenic crisis requiring acute treatment throughout the disease course. There was no difference concerning age of onset (26.1% EOMG vs. 19.5% LOMG, *p* = 0.320). In total, 41 (19.3%) patients required hospital admission due to MG exacerbations, 20 EOMG (16.1%) and 21 LOMG (25.6%) (*p* = 0.335). Regarding the treatment regimens, pyridostigmine was offered to 93.9% of patients, with significantly more EOMG patients receiving treatment (*p* = 0.024). The median highest dose of anticholinesterase was 300.0 (240.0–360.0), which was higher in EOMG compared to LOMG (300.0 versus 240.0, *p* < 0.001). Acute treatment modalities comprised human immunoglobulin (30.7%) and plasmapheresis (6.1%). Steroids were used in 74.5% of patients, with 15.6% requiring prednisolone as part of maintenance treatment. Treatment-related side effects were the most common reason for choosing a steroid-sparing agent (82.0%). From these, hypertension, hypercholesterolemia, diabetes mellitus and malignancies were more common in LOMG, while osteoporosis and dyspepsia were more frequent in EOMG (*p* < 0.001) (Figure 2).

Most patients were switched to steroid-sparing agents, with the most common first-line choice being azathioprine (45.8%). Rituximab was used as a first-line option in 4 patients (1.9%): 2 anti-MuSK-associated MG cases, 1 with comorbid autoimmune disease and 1 EOMG case with an aggressive course. There was no significant difference between EOMG and LOMG regarding first-line options. Second-line immunotherapy was required in 26.9%, mainly for improving disease control (85.8%). Treatment-related adverse events motivating a therapy switch to second-line agents (3.8%) included hepatitis (2.4%), repeated infections (0.9%) and pancytopenia (0.5%). The most common second and third-line agents were methotrexate (12.3%) and rituximab (6.1%), respectively. Tocilizumab (0.5%) and efgartigimod (0.5%) were only used as third-line alternatives in EOMG cases. Thymectomy was performed in 81 (38.2%) patients, more frequently in EOMG (81.5%) than LOMG (18.5%). The median time to thymectomy was 1.0 (1.0–3.0) years. Thymoma was identified in 30 (14.2%), most commonly of type 2B (53.3%), while hyperplasia was present in the remaining patients. Although more thymomas being identified in EOMG (56.7% vs. 43.3%), this difference was not statistically significant.

## Discussion

This study underscores the importance of considering the age of MG onset and comorbidities in treatment selection.

Overall, our MG cohort follows what is described in the literature for EOMG concerning female predominance and tendency toward generalized disease (1, 6). Thymomas were more frequently found in LOMG cases (*p* < 0.001), which is consistent with the literature (7). However, patients proposed for thymectomy were already the ones with an increased probability of having a thymoma based on imaging studies, which could introduce bias.

In our cohort, most MG patients had at least one comorbidity (approximately 59%), these being more frequent in LOMG cases. LOMG had significantly more comorbidities, particularly comorbidities associated with increased cardiovascular risk (arterial hypertension, dyslipidemia, and diabetes mellitus), consistent with previous reports (4). However, we could not exclude that these resulted from older age and not disease type. A previous study compared age-and sex-matched groups of EOMG and LOMG and found no significant differences, consistent with comorbidities probably resulting from the cumulative effect of increased aged than with MG type (5). Some conditions like dyslipidemia and hypertension may be particularly bothersome in MG since some treatment options (statins and beta-blockers) might worsen MG symptoms (8).

Steroid-related adverse effects motivating treatment change were frequent, consistent with the general recommendations for corticotherapy. However, the profile of side effects differs between LOMG and EOMG. Steroid-treated LOMG more frequently developed malignancies and the same comorbidities that were already more frequent in this subgroup (arterial hypertension, hypercholesterolemia, diabetes mellitus). This effect might be explained by age, as previously addressed. In the EOMG group, osteoporosis and dyspepsia were more frequent. Steroid-induced osteoporosis mainly affects individuals 20–45 years, consistent with our findings (9, 10). This probably results from the cumulative effect of corticotherapy for several years, since the mean dose was not significantly different between groups.

Immunosuppression is generally associated with favorable outcomes in MG, irrespective of the age of onset (11). In most cases from our study, steroid sparing agents were introduced first, and the dose of steroids progressively tapered while a steroid-sparing agent was introduced. The choice of steroid-sparing agents followed what is recommended in the literature, with the most frequent options being azathioprine, mycophenolate mofetil and methotrexate (1, 8, 12). MMF has been suggested as an alternative first-line steroid-sparing agent, due to its favorable profile (13). The results from our practice, with AZA as the commonest option, are in line with recently published guidelines (14). Anti-CD20 antibody therapy (rituximab) was generally reserved to more severe or refractory cases. These findings are consistent with other studies showing that the therapeutic management does not seem to differ between EOMG and LOMG, despite the latter having more comorbidities (11, 15). Rituximab has also been shown to be a safe and effective treatment in late-onset cases of aggressive generalized MG (16). Regarding other monoclonal antibodies, eculizumab and tocilizumab were used in 1 EOMG patient each, which is statistically non-significant. Both treatments have been shown to be safe and effective in patients with later age of onset (17, 18). Overall, there was a tendency toward late-onset forms requiring fewer drugs and thus being less frequently treatment-refractory, confirming findings from a recent study (6).

This study has several limitations that must be addressed. First, its retrospective design based on clinical records with a relatively small sample size. Second, we opted to use the age of 50 as a cut-off to define EOMG and LOMG, but there are other cut-off values proposed in the literature (19). Moreover, we have not considered the subgroup of very-late onset forms that is increasingly recognized (20). We have not specifically addressed the efficacy of different treatment modalities based on the MGFA score, as the purpose of this study was to specifically study treatment modalities and respective side effects in EOMG and LOMG. In the future, doing a longitudinal study with this purpose would be interesting.

MG is a complex neuroimmunological disorder whose treatment implicates taking into account patient comorbidities and strategies to avoid treatment-related adverse events. The growing subgroup of LOMG poses further challenges to neurologists handling this disorder. Our study describes the similarities in treatment modalities between EOMG and LOMG and the differences in steroid-related adverse events between each group.

## Data availability statement

The raw data supporting the conclusions of this article will be made available by the authors, without undue reservation.

## Ethics statement

The studies involving humans were approved by Comissão de ética do Centro Hospitalar Universitário de Santo António. The studies were conducted in accordance with the local legislation and institutional requirements. The ethics committee/institutional review board waived the requirement of written informed consent for participation because the retrospective study was based on information from clinical records.

## Author contributions

JM: Conceptualization, Formal analysis, Methodology, Writing – original draft, Writing – review & editing. JF: Conceptualization, Formal analysis, Methodology, Writing – original draft. ML: Conceptualization, Formal analysis, Methodology, Writing – original draft. AS: Writing – review & editing. RS: Writing – review & editing. AM: Writing – review & editing. ES: Conceptualization, Writing – review & editing.
